# Determinants of periodontitis according to the immunological and virological profiles of HIV-infected patients in Yaoundé, Cameroon

**DOI:** 10.1186/s12903-020-01353-7

**Published:** 2020-12-11

**Authors:** Joseph Fokam, Buolikeze Kuoh Nji Geh, Samuel Martin Sosso, Desire Takou, Ezechiel Semengue Ngufack, Alex Durand Nka, Anne-Cecile Z.-K. Bissek, David Mindja Eko, Alexis Ndjolo

**Affiliations:** 1 Chantal BIYA International Reference Centre for Research on HIV/AIDS Prevention and Management (CIRCB), Melen Road, PO Box 3077, Yaoundé, Cameroon; 2 Faculty of Medicine and Biomedical Sciences (FMBS), University of Yaoundé I, Yaoundé, Cameroon; 3 National HIV Drug Resistance Surveillance and Prevention Working Group (HIVDR-WG), Ministry of Public Health, Yaoundé, Cameroon; 4 Division of Health Operational Research, Ministry of Public Health, Yaoundé, Cameroon

**Keywords:** HIV, Periodontitis, CD4, CD8, Viral load

## Abstract

**Background:**

HIV infection is associated to different oral manifestations (including periodontal diseases), which have decreased with the advent of antiretroviral therapy (ART). Yet, the occurrence of periodontitis is still consistent among patients with HIV living in sub Saharan-Africa, with limited evidence on the driven factors and mitigating measures in these settings. We aimed at evaluating the occurrence of periodontitis and its associated immunological and virological factors in patients with HIV living in Yaoundé, Cameroon.

**Methods:**

We included 165 (44 ART-naïve and 121 ART-experienced) patients > 18 years old attending the Yaoundé Central Hospital and the Chantal BIYA International Reference Centre, from January-April 2018. The periodontal status was assessed by measuring the clinical attachment loss, periodontal pocket depth, plaques index and gingival bleeding index. CD4+/CD8+ cells and viremia were measured using the fluorescence-activated cell sorting method (FACS Calibur) and the Abbott m2000 RT HIV-1 RNA kit respectively. A standard-questionnaire concerning participants’ medical records and oral hygiene methods was filled. Data was analyzed and *p* < 0.05 considered statistically significant.

**Results:**

There was a significantly high prevalence of periodontitis in the ART-naïve (53.2%) compared to the ART-experienced group (37.3%), with a twofold increased risk of the ART-naïve population presenting with periodontitis than the ART-experienced population (OR 2.06, *p* = 0.03). More importantly, ART-naïve, patients with CD4 < 200 cells presented with higher risk of having periodontitis compared to those with higher CD4-values, with a threefold difference (OR 3.21). Worth noting, males presented with a higher risk of having clinical attachment loss (OR 6.07). There was no significant association between the occurrence of periodontitis and the CD8 (*p* = 0.45) or viremia (*p* = 0.10).

**Conclusion:**

In the Cameroonian context, a considerable number of adults infected with HIV suffer from periodontitis regardless of their treatment profile. Nonetheless, ART-naïve patients have a higher risk, indicating the protective role of ART. Interestingly, severely immune-compromised patients and men are vulnerable to periodontitis, thereby highlighting the need for clinicians to refer patients for regular periodontal screening especially male patients and those with low CD4. Such measures could greatly improve the quality of life of the population living with HIV in Cameroon.

## Background

The Human immunodeficiency Virus (HIV) remains a global health problem with over 36.7 million infected people in 2016 according to WHO [1]. Worth noting, sub-Saharan Africa carries about 70% of the global burden and the prevalence of HIV in Cameroon was 3.4% in 2018 [2]. HIV is a retrovirus, whose infection is characterized by a combination of clinical manifestations which are caused by the infection directly and killing of CD4+ lymphocytes, thus decreasing the natural defense mechanism leading and severe immunodeficiency.

The introduction of Highly Active Antiretroviral Treatment (HAART) has greatly increased the life expectancy of patients living with HIV. Yet HIV infection is still associated to different oral manifestations [3] such as oral hairy leukoplakia, oral candidiasis, Kaposi's sarcoma, non-Hodgkin lymphoma, as well as periodontal diseases such as linear gingival erythema, necrotizing ulcerative gingivitis, necrotizing ulcerative periodontitis and possible exacerbation of pre-existing periodontal conditions.

HIV is therefore a risk factor for periodontitis, as immunological changes in HIV infection may alter the patient’s ability to respond appropriately to infection, including those due to sub-gingival bacterial [4]. There are various types of cells found in the gingival fluid including Type 1 and Type 2 helper cells whose imbalance in cytokine production can induce bone resorption hence clinical attachment loss (CAL) [5]. Pro-inflammatory cytokine such as Interlukin-1 (IL-1) and alpha-tumor necrosis factor (TNf-α) stimulate the production of osteoprotegerin ligand by osteoblastic cells which later differentiate into precursor osteoclast and then osteoclast leading to bone resorption, hence periodontitis in patients [6]. To reduce the risk of opportunistic infections, including oral manifestations, international medical associations developed guidelines for starting antiretroviral treatment despite the CD4 cell counts in these patients known as the test and treat approach implemented in Cameroon since 2016 [7]. Importantly, patients with HIV may present distinct immunological/virological profiles and different oral manifestations [8]. The immunological parameter usually used for this evaluation is the CD4/CD8 T-lymphocyte counts in peripheral blood and plasma viral load, which appear to play an important role in the progression of periodontal disease and the course of HIV-infection [9].

Despite the high prevalence of HIV infection in Cameroon [2], little or no reports about the periodontal status of HIV-infected Cameroonians have been published. With the aim to identify high risk populations so as to set up better preventive measures, the present study tested the hypothesis that periodontal disease in persons living with HIV was significantly related to low CD4/CD8 ratio and an increase in plasma viral load.

## Methods

### Study design

A prospective, cross sectional and analytical study was conducted among people living with HIV at the Chantal BIYA International Reference centre for prevention and management of HIV/AIDS (CIRCB), in Yaoundé–Cameroon, from January 2018 through April 2018. Our sample size was calculated using the following formula:

$${\text{N}} = \frac{{{\text{Za}}^{2} \times {\text{P}}\left( {1 - {\text{P}}} \right)}}{{{\text{d}}^{2} }}$$

With “Za” being the 95% confidence interval (CI) set at 1.96, “P” being the previous prevalence of periodontitis in the same city and study population which was (76.2%) and “d” being the error rate set at 5% (0.05); resulting in a minimum sample size “N” = 278.6, rounded-up to 279 study participants.

According to the Center for Disease Control (CDC) case definition, periodontitis could be defined as moderate or severe, severe when two or more interproximal sites (not on the same tooth) attained with CAL ≥ 6 mm and at least one interproximal site with pocket depth ≥ 5 mm, while moderate periodontitis was defined as two or more interproximal sites (not on the same dental organ) with CAL ≥ 4 mm or two or more interproximal sites with pocket depth ≥ 5 mm (also not on the same dental organ) using the CDC population-based surveillance for periodontitis.

Participants were enrolled at the medical units of CIRCB and at the approved Treatment center for HIV of the Yaoundé Central Hospital. Following the study information notice, participants were enrolled based on the following inclusion criteria: adult HIV-infected patients both ART-naïve and ART-experienced patients ≥ 21 years, who gave their consents and were seen at our study site. Briefly, ART-naïve patients are those who have never been on antiretroviral treatment with respect to their HIV status, and ART-experienced patients are those who are on antiretroviral treatment at the time of the study. We excluded patients with diabetes, patients not monitored at any of our study sites, hospitalised HIV-infected patients and HIV pregnant women.

### Clinical examination

A self-administered questionnaire was used to obtain socio-demographic characteristics of the participants, medical treatment records (use of ARV drugs or not) and oral hygiene competence (brushing frequency). Immunological values (CD4, CD8, CD4/CD8 ratio, and viral load) of participants were gotten from blood samples assessed in the laboratory. All clinical examinations for periodontitis were carried out by a single examiner (lead author) using WHO periodontal probe (WHO 2002) to ensure consistency of measurements. The WHO periodontal probe with 0.5–3.5–5.5–8.5–11.5 mm graduations was positioned parallel to the long axis of the tooth at each site and measurements were rounded to the lower whole millimeter. Periodontal examination included a number of parameters among which the periodontal pocket depth (PPD in mm) and gingival recession (GR) were recorded at six sites per tooth (mesio-buccal, disto-buccal, buccal, lingual, disto-lingual and mesio-lingual/palatal); Clinical Attachment loss (CAL in mm) was calculated as the sum of periodontal pocket depth (PPD) and gingival recession (GR) per site of the dental organ. The CAL was used to determine the severity of periodontitis in these patients.

### Assessment of immunological (CD4/CD8) and virological parameters

Blood samples were collected from HIV-infected patients and analyzed at the clinical diagnostic laboratory of the CIRCB for CD4/CD8 and plasma viral load. Briefly, CD4/CD8 counts were performed by flow cytometry of Becton Dickson’s “FACS Calibur” according to the manufacturers instructions (https://www.bdbiosciences.com/documents/BD_FACSCalibur_Brochure.pdf); plasma viral load was performed by Real Time PCR on the ABBOTTm2000RT platform as per manufacturer’s instructions (www.abbottmolecular.com/products/infectious-disease/realtime-pcr/hiv-1-assay).

### Statistical analysis

Data was entered into Microsoft excel 2013 and analyzed using the statistical software Epi Info version 7.2.2.6 with results presented as mean, standard deviation, median, quartiles, frequencies and percentages. Bivariate analysis was done using Fischer’s exact test and Chi-square test. Multivariate analysis was done using the logistic regression model. All p values < 0.05 were considered statistically significant.

### Ethical considerations

The study protocol was approved by the Institutional Ethics Committee for Research of the Faculty of Medicine and Biomedical Sciences (Reference: 0063/UY1/FMSB/VDRC/CSD). Written informed consent was provided by each study participant, data were collected under strict confidentiality and respect of patient privacy. Dental consultations were done free of charge and results of plasma viral load and CD4 were freely returned to patients for the personal clinical benefit and wellbeing in both HIV management and dental care.

## Results

### Characteristics of the study population

A total of 165 participants were enrolled including 108(65.45%) females and 57 (45.55%) males, 44 were ART-naïve patients and 121 were ART-experienced. In the ART-naïve population we had 17 males and 27 females while in the ART-experienced population, there were 40 males and 81 females. The mean ages in both groups were 39.23 ± 11.5 (ART-naive) and 43.48 ± 10.2 years (ART-experienced), seen in Table 1. The majority of our study population were traders.

**Table 1:** Age distribution in study population

Age range	Naïve n (%)	ART experience n (%)	Total population n (%)
20–<30	8 (18.18%)	9 (7.44%)	17 (10.30%)
30–<40	16 (36.36%)	35 (28.93%)	51 (30.91%)
40–<50	12 (27.27%)	42 (34.74%)	54 (32.73%)
50–<60	5 (11.36%)	27 (22.31%)	32 (19.39%)
60–<70	3 (6.82%)	8 (6.61%)	11 (6.67%)
Total	44 (100%)	121 (100%)	165 (100%)

Population characteristics	Naïve	ART experienced	Total population
Residence: Rural	12	43	55
Urban	32	78	110
Religion: Atheist	0	1	1
Christian	38	116	154
Muslim	6	4	10
Region: Adamawa	1	3	4
Central Africa Republic	1	0	1
Center	22	52	74
East	0	2	2
Far north	1	1	2
Littoral	2	5	7
North	1	2	3
North west	3	7	10
South	6	11	17
South west	0	3	3
West	7	35	42
Profession			
House wife	6	27	33
Independent	0	1	1
Private sector	2	11	13
Public sector	10	34	44
Retired	0	7	7
Student	4	1	5
Trader	22	36	58
Unemployed	0	4	4
Marital status			
Divorced	4	10	14
Married	12	56	68
Single	26	44	70
Widow	2	11	13
Educational level			
None	0	2	2
Primary	10	18	28
Secondary	22	78	100
Superior	12	23	35

The most frequently used therapy registered was Tenofovir (TDF) + lamivudine (3TC) + Efavrienz (EFV) for 49.27% of the participants and the least frequent therapy was TDF + 3TC + lopinavir boosted with ritonavir (LPV/r) for only 0.61% of our study participants. Four (3.03%) patients had undocumented treatment history as indicated in Table 2.

**Table 2:** Distribution of ART in the ART-experienced population

ART	Frequency	Percentage
ABC/3TC/LPVr	2	1.73
Tenlam LPV/r	1	0.86
Tenlam-ATV/r	4	3.45
TenlamE	78	67.24
TenlamN	11	9.48
ZidolamN	20	17.24
Total	116	100

TenlamE, Tenofovir+ lamivudine+ Efavirence; TenlamN, Tenofovir+ lamivudine+ Niverapine; ART, antiretroviral therapy; ABC/3TC/LPVr, Abacavir+lamivudine+ Lopinavir/ritonavir; ZidoamN, Zidovudine+ lamivudine+ Niverapine

**Fig. 1:**
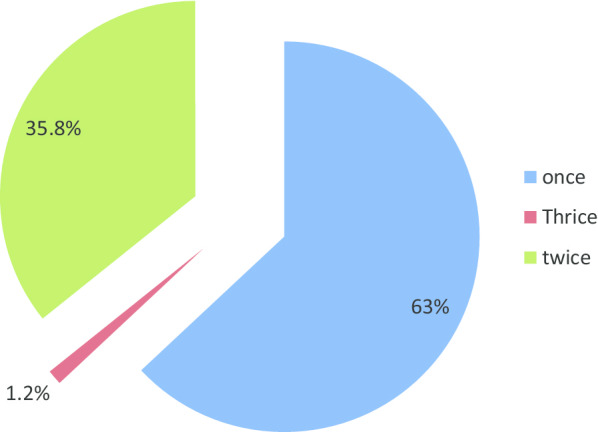
Daily brushing frequency distribution in the study population

Majority (63.03%) of the study population had a brushing frequency of once daily (morning before breakfast) which was the inappropriate time to brush, followed by those with a brushing frequency of twice daily (mornings before breakfast and night before sleeping), seen in Fig. 1.

Our study participants were mainly made up of former smokers and alcohol consumers (who have stopped smoking/alcohol for about 5 years and above by the moment of the study period).

### Periodontitis in the study population

The overall prevalence of periodontitis was 39.4%. In the ART-naïve population, the prevalence of Periodontitis was at 53.2% (23/44) versus 34.7% (42/121) in the ART-experienced (*p* = 0.03).Thus naïve patients had two times higher risk of presenting with periodontitis than the ART-experienced (Table 3).

**Table 3:** Distribution of periodontitis in study population

Group	PD present (n)	PD Absent (n)	Odds Ratio	*p* value
Naïve	23	21	2.06	0.03
ART	42	79		
Total	65	100		

PD, periodontitis; ART, antiretroviral therapy

**Fig. 2:**
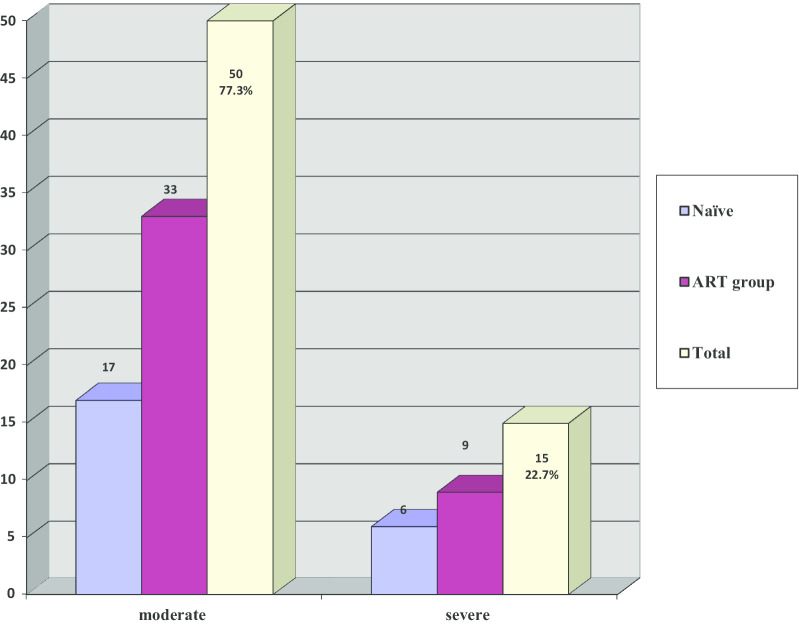
Distribution of periodontitis in the study population

Among patients with periodontitis (in both ART-naïve and ART-experienced groups), 50 (77.3%) of the cases had moderate periodontitis and 15 (22.7%) had severe periodontitis seen in Fig. 2. In the ART-experienced population, there was no significant relationship between the prevalence of periodontitis and the various ART regimens (*p* = 0.10) as reported in Table 4.

**Table 4:** Distribution of periodontitis according to the ART regimen

Type of ART	PD present		Odds ratio	*p* value
	Yes	No		
1st line	40	70	0.45	0.10
2nd line	2	5		
Total	42	75		

PD, periodontitis; ART, antiretroviral therapy

In the ART-naïve population, there was three times higher risk of periodontitis for naïve patients. In the ART-experienced group, there was an associated risk with lower CD8 (< 300) and the occurrence of periodontitis (OR 1.85).

The mean (± SD) viral load of the total population was 1.9 log (2.24) copies/ml.in the ART-naïve and ART-experienced group mean (± SD) viral load was 3.31log (1.16) and 1.39 (0.967) copies/ml respectively.

No significance was seen in the association of viral load and the occurrence of periodontitis in both ART-naïve and ART-experienced groups (Table 5).

**Table 5:** Distribution of periodontitis according to immunological and virological markers

	Naïve				ART group			
	PD present	PD absent	Odds ratio	*p* value	PD present	PD absent	Odds ratio	*p* value
CD4cells/mm								
< 200	15	7	3.21	0.06	6	14	0.74	0.38
> 200	8	12			36	62		
CD8								
< 300	1	2	0.4	0.43	2	2	1.85	0.45
> 300	22	17			40	74		
CD4/CD8								
< 1	20	18	0.37	0.38	31	66	0.43	0.06
> 1	3	1			11	10		
Viral load log								
> 3log	14	14	1	0.76	5	18	0.45	0.10
< 3log	1	1			36	58		

ART, antiretroviral therapy; PD, periodontitis

Multivariate analysis was done associating gender (male/female), age, CD4, CD8, ART-naïve patients with clinical attachment loss in the study population.

In this analysis there was a statistical significance (*p* = 0.0008) for Gender (male) and age. The associations between the occurrence of periodontitis with a low CD4 and age were seen to be higher in naïve patients.

Of relevance, age was significantly associated with attachment loss, suggesting the potential role of age as a risk factor of periodontitis, as shown in Table 6.

**Table 6:** Logistic regression of variables associated with clinical attachment loss

	Odds ratio	95% CI	Z-statistic	*p* value
CD4	1.0005	0.9989	0.6378	0.5236
Sex (male/female)	6.077	2.1148	3.3507	0.0008*
Age	1.0969	1.049	4.0651	0*
Naïve (true/false)	1.2535	0.5009	0.4828	0.6293
CD8	1.0001	0.9997	0.2601	0.7948

ART, antiretroviral therapy

*Significant

## Discussions

The present study evaluated immunological and virological determinants of periodontitis in patients with HIV. 65% of the population were females, the male to female ratio is similar to the gender distribution reported in the general population of people living with HIV in Cameroon as well as in other studies, thus supporting the vulnerability of women to HIV and the possible representatives of our findings to the target population of adults living with HIV in Cameroon [10, 11]. The median CD4 in the ART-naïve (176 cells/mm^3^) was lower than in the ART-experienced (443 cells/mm^3^) patients but both appeared to be lower as compared to findings reported by Khammissa et al. [11] where the median CD4 in ART-naïve patients was higher than in the ART-experienced population. This is likely attributed to the late diagnosis of HIV in their study population. On the other hand, higher levels of viral load in the ART-naïve compared to the ART-experienced groups basically reflects the selective pressure of HAART among the ART-experienced patients.

The overall prevalence of periodontitis (39.4%) was similar to that in other studies carried out in HIV-infected patients (with prevalence 36.11% and 34% respectively) [12, 13]. Worth noting, the significantly higher rate of periodontitis among ART- naïve compared to ART-experienced patients (*p* = 0.03) supports the fact that naïve patients have a higher risk of presenting with periodontitis. This in turn highlights the protective role of ART in the current era of “test all” or “test and treat” in resource limited settings like Cameroon. However, ART regimen did not specifically alter the occurrence of periodontitis as previously supported by Fricke et al. [14]. Furthermore Fricke et al. reported an association between the duration of infection and periodontitis which was not considered in our present study.

Subsequently there was no significant association with low CD4 (CD4 < 200 cells/mm^3^) and the occurrence of periodontitis in both ART-naïve and ART-experienced populations. These results are in agreement with that of Umeizudike et al. [10], who did not find any statistical association as well (*p* = 0.623). It’s worth noting that ART-naïve population with CD4 cells less than 200 had three times higher risk of having periodontitis than the ART group. This is similar to the findings of Vernon et al. [15] in which those with CD4 cells less than 200 had two times higher risk of presenting with periodontitis than those with CD4 cells greater than 200 (*p* = 0.001). These concordant evidences therefore support that having CD4 cells less than 200 is a strong predictor of periodontitis in population living with HIV.

There was no significant associations with CD4 cells < 200/mm^3^ and other periodontal clinical parameters. This observation was concordant with a previous study reporting no significant association between CD4 cells < 200/mm^3^ and clinical periodontal parameters [16] A study carried out in India showed a statistically significant association between the immune status and periodontitis, thus calling for context specific investigations in delineating such disparities [13, 17].

Our study revealed men had a significantly higher risk of presenting with periodontitis than women (*p* = 0.0008), as supported by several previous findings in the same population on different countries [18, 19]. This could be explained by the fact that men have a less positive attitude towards their oral health than women, and men generally attend clinics lately as compared to women thus favoring advanced disease conditions in the frame of late diagnosis. Age was significantly associated with CAL, suggesting the potential role of age as a risk factor of periodontitis, similar to the findings of Umeizudike where those with age > 35 years where significantly associated with periodontitis [18].

Our study was limited by the small sample size of the ART-naïve population. Second there was lack of follow-up for these patients. Third, characterization of the oral microbiome in HIV-infected and HIV-uninfected subjects with their ART status was not done and could have been worth evaluating. Hence, studies covering these aspects should be carried out especially for the ART-naïve populations.

**Fig. 3:**
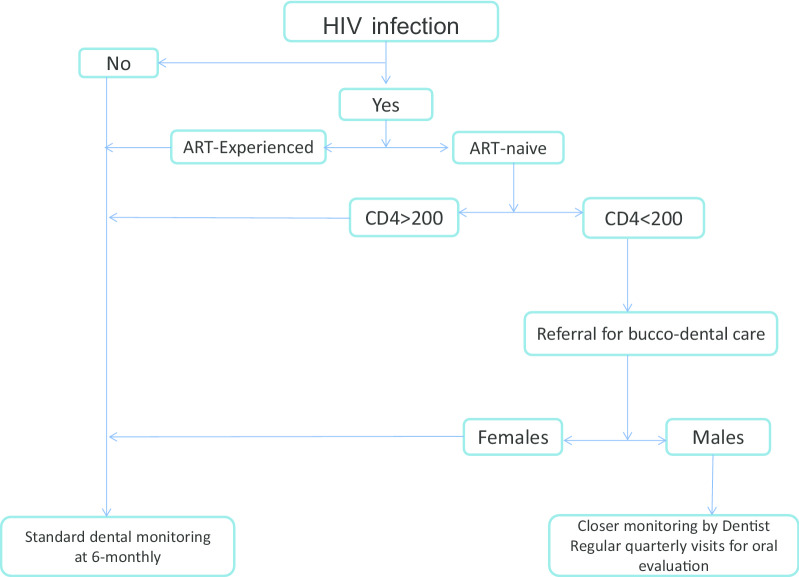
Proposed treatment algorithm

Overall, our evidence-based findings suggest the following algorithm for an improved management of oral health in HIV highly-burdened resource limited settings, Fig. 3.

## Conclusion

In our study, ART-naïve HIV-infected patients especially those of male gender with severe immunodeficiency (CD4 cells < 200/mm^3^) had a significantly higher risk of periodontitis. Thus, priority intervention towards a better prevention of periodontitis among people living with HIV requires the need for clinicians to ensure systematic referral for periodontal screening (bucco-dental checkups) for every HIV-infected male or immunodeficiency patient.

## Data Availability

The datasets used and/or analyzed during the current study are available from the corresponding author on reasonable request.

## Abbreviations

AIDS: Acquired Immune Deficiency Syndrome
ART: Anti-retroviral treatment
ARV: Anti-retroviral
CAL: Clinical attachment loss
CD4: Cluster differentiation cells
CDC: Center for Diseases Control and Prevention
CIRCB: Centre International de Reference Chantal Biya
GR: Gingival recession
HAART: Highly active anti-retroviral therapy
HIV: Human immunodeficiency virus
IL: Inter-leukin
mm: Millimeter
mm^3^: Cubic millimeter
PCR: Polymerized chain reaction
PD: Periodontal disease
PGE: Prostaglandin
PPD: Periodontal pocket depth
TNF: Tumor necrosis factor
WHO: World Health organization
